# Identification of a novel *GNAS* mutation in a case of pseudohypoparathyroidism type 1A with normocalcemia

**DOI:** 10.1186/s12881-018-0648-z

**Published:** 2018-07-30

**Authors:** Xiao-dan Long, Jing Xiong, Zhao-hui Mo, Chang-sheng Dong, Ping Jin

**Affiliations:** 1 Department of Endorcrinology, The third Xiangya Hospital Central South University, Tongzipo Road, 410007 Changsha, Hunan Province People’s Republic of China; 2 Department of Anesthesia, The affiliated Tumor Hospital of Xiangya Medical School of Central South University, Changsha, 410007 Hunan China

**Keywords:** Pseudohypoparathyroidism type 1A, *GNAS* gene, Stimulatory G-protein alpha subunit, Mutation

## Abstract

**Background:**

Pseudohypoparathyroidism type 1A (PHP1A) is a rare genetic disease primarily characterized by resistance to parathyroid hormone along with hormonal resistance and other features of Albright hereditary osteodystrophy (AHO). It is caused by heterozygous inactivating mutations in the maternal allele of the *GNAS* gene, which encodes the stimulatory G-protein alpha subunit (Gsα) and regulates production of the second messenger cyclic AMP (cAMP). Herein, we report a case of of PHP1A with atypical clinical manifestations (oligomenorrhea, subclinical hypothyroidism, and normocalcemia) and explore the underlying genetic cause in this patient.

**Methods:**

Blood samples were collected from the patient, her family members, and 100 healthy controls. The 13 exons and flanking splice sites of the *GNAS* gene were amplified by PCR and sequenced. To further assess whether the novel mutation resulted in gain or loss of function of Gsα, we examined the level of cAMP activity associated with this mutation through in vitro functional studies by introducing the target mutation into a human *GNAS* plasmid.

**Results:**

A novel heterozygous c.715A > G (p.N239D) mutation in exon 9 of the *GNAS* gene was identified in the patient. This mutation was also found in her mother, who was diagnosed with pseudopseudohypoparathyroidism. An in vitro cAMP assay showed a significant decrease in PTH-induced cAMP production in cells transfected with the mutant plasmid, compared to that in the wild-type control cells (*P* < 0.01), which was consistent with loss of Gsa activity.

**Conclusion:**

We identified a novel *GNAS* mutation that altered Gsα function, which furthers our understanding of the pathogenesis of this disease. Screening for *GNAS* mutations should be considered in suspected cases of PHP1A even if the classical signs are not present.

**Electronic supplementary material:**

The online version of this article (10.1186/s12881-018-0648-z) contains supplementary material, which is available to authorized users.

## Background

Pseudohypoparathyroidism (PHP) is a rare heterogeneous disease characterized by hypocalcemia and hyperphosphatemia due to resistance to parathyroid hormone (PTH) in target organs [1]. Its prevalence is estimated to be 0.34/100000 in Japan [2] and 1.1/100000 in Denmark [3]. PHP is classified into different types based upon distinct biochemical profiles and clinical manifestations, including PHP1A(OMIM 103580), PHP1B (OMIM 603233), PHP1C (OMIM 612462) and PHP2 (OMIM 203330) [4, 5]. PHP1A and PHP1B are the most prevalent subtypes, with similar prevalence rates (48% vs 46%) according to a recent study by Elli et al [6]. PHP1A is characterized by target organ resistance to parathyroid hormone (PTH) and features of Albright’s Hereditary Osteodystrophy (AHO) such as round face, short stature, subcutaneous calcifications and brachydactyly, whereas PHP1B classically presents as hormone resistance limited to PTH without AHO signs. PHP1A is caused by heterozygous inactivating germline mutations in the guanine nucleotide-binding protein α-stimulating polypeptide (*GNAS*) gene located at chromosome 20q13.3 [7]. PHP1A is inherited as an autosomal-dominant trait under imprinting caused by genetic alterations at the maternal allele.

The protein encoded by the *GNAS* gene is the α-subunit of the stimulatory GTP binding protein (Gsα), which is involved in a large number of signal transduction pathways for multiple hormones via the stimulation of adenylyl cyclase through production of cyclic AMP (cAMP) [8]. To date, more than 400 inactivating *GNAS* mutations have been reported, including frameshift, missense, nonsense, splice-site mutations, in-frame deletions or insertions, and whole or partial gene deletions [6], and most of them lead to a truncated protein. These mutations are scattered throughout the whole coding region of *GNAS* and only a recurring 4-bp deletion in exon 7 (p.D190MfsX14) has been considered a mutational hot spot [9]. In general, no genotype–phenotype correlation has been found for the inactivating *GNAS* mutations. However, a temperature-sensitive Gsα mutant (p.A366S) that causes testotoxicosis has been described in previous studies [10, 11].

Elevated levels of PTH and serum phosphate as well as a low level of serum calcium are the hallmark features of PHP1A. Here, we report a novel heterozygous *GNAS* mutation in a female patient who initially presented to us with oligomenorrhea and was subsequently diagnosed with PHP1A despite characteristics of normocalcemia and normophosphatemia.

## Methods

### Patients

**Fig. 1:**
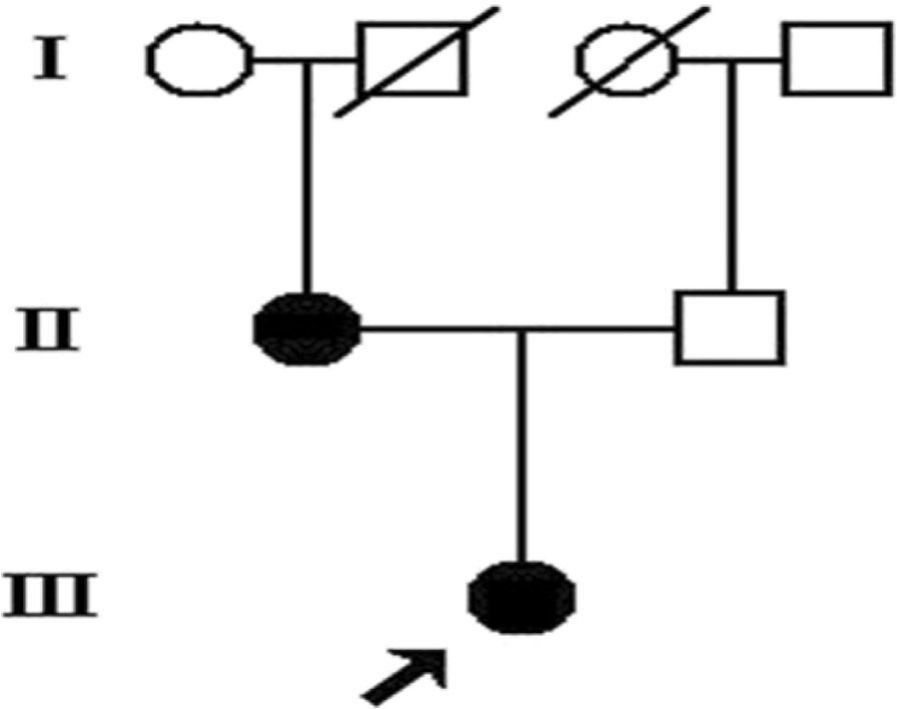
Pedigree of the studied family; the proband is indicated by the arrow

**Fig. 2:**
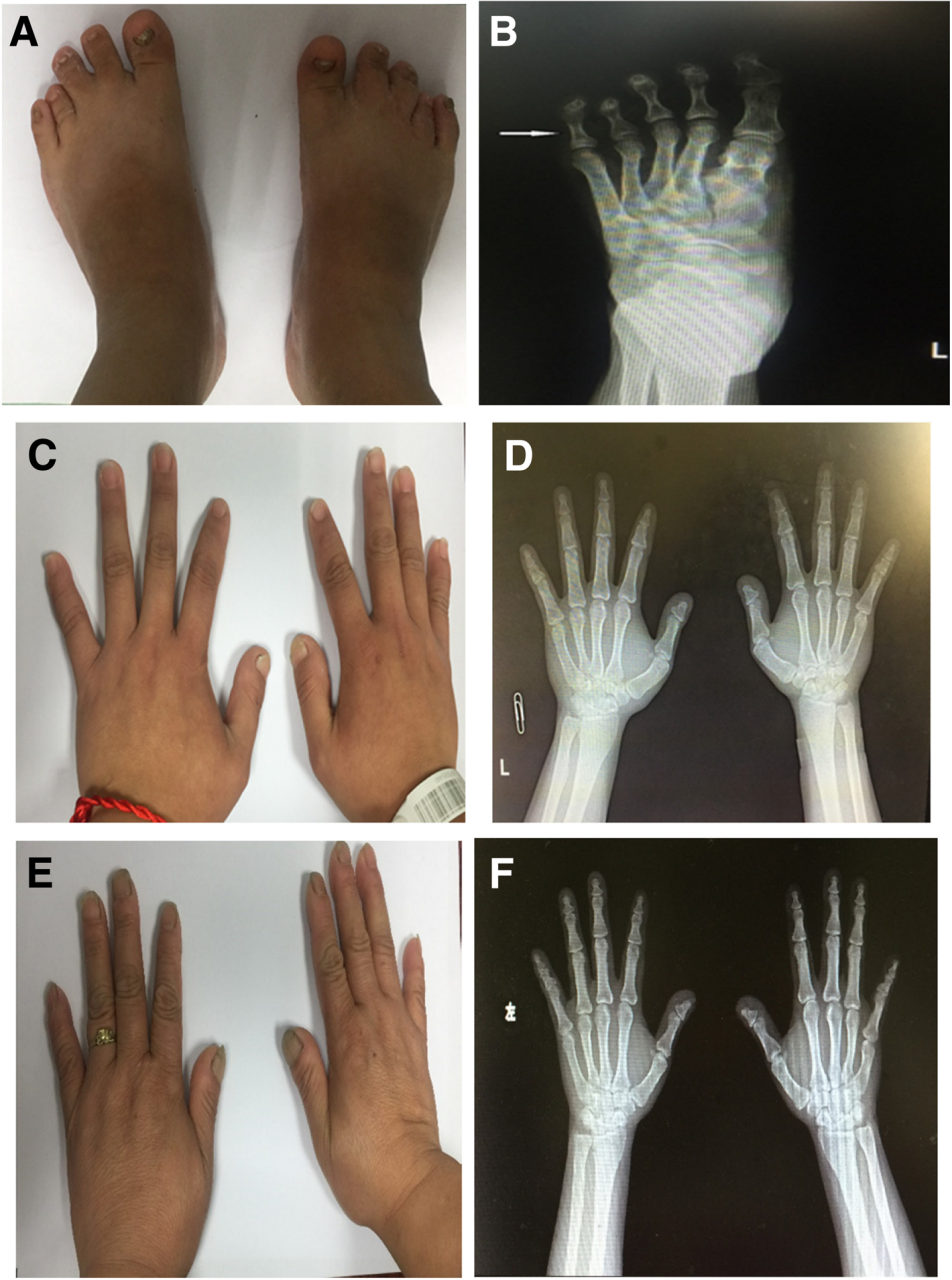
(**a**) Photograph of the patients feet and (**b**) X-ray of the left feet showing brachymetatarsia. (**c**) Photograph of the patient’s hands and (**d**) X-ray of her hands (**d**) showing brachydactyly, typical of Albright’s osteodystrophy. (**e**) Photograph of the patient’s mothers hand and (**f**) X-ray of her hands (**f**) also showing slight brachydactyly similar to her daughter

We studied a family affected by PHP (Fig. 1). The proband was an 18-year-old girl who was referred to our hospital in 2016 because of 6 years of irregular menstruation and 2 years of thyroid dysfunction. She had first menstruation at the age of 12 years, and thereafter had oligomenorrhea with only one to two menstrual periods per year. Two years prior to presentation, she was diagnosed with subclinical hypothyroidism with an elevated thyroid-stimulating hormone (TSH) level of 16.1 μIU/mL (reference range [RR], 0.5–4.3 μIU/mL) and a normal free thyroxine (FT4) level. L-thyroxine replacement treatment was started, but her TSH level remained high (10.7~ 26.09 μIU/mL) despite the use of increasing doses of L-thyroxine from 50 μg to 200 μ q.d. On physical examination, she showed features of AHO: round face, short stature, obesity (body weight = 60 kg, height = 150 cm, body mass index = 26.7 kg/m^2^), brachymetatarsia (Fig. 2a,b) and brachydactyly (Fig. 2c,d), without subcutaneous or intracranial calcifications. Laboratory tests revealed elevated serum PTH, high-to-normal serum phosphate, normal serum calcium, and 25-hydroxyvitamin D3 levels (Table 1). A low 24-h urinary calcium measurement of 0.83 mmol/day (RR, 2.5–7.5 mmol/day) was recorded, whereas renal tubular reabsorption of phosphate (TmP/GFR) was 1.05 mmol/L (RR, 0.81–1.45 mmol/L). Other investigations revealed that she had subclinical hypothyroidism with a high serum TSH level and normal FT_4_ level and was negative for thyroid autoantibodies. Her serum follicle-stimulating hormone (FSH) and luteinizing hormone (LH) levels were a slightly elevated, and her estradiol level was low (Table 1), resembling hypergonadotropic hypogonadism. She had a normal basal cortisol level and increased adrenocorticotropic hormone (ACTH) concentration (Table 1), but clinical signs of adrenal deficiency were not observed. Her growth hormone (GH) level, blood glucose level, and islet function were all normal (Table 1). Osteopenia of the lumbar spine with a Z score of − 1.5 was revealed on a bone mineral density test. Based on these clinical and biochemical findings, she was diagnosed with PHP1A. Subsequently, the patient was prescribed calcitriol (0.5 μg/bid), L-thyroxine (75 μg/q.d), and estrogen-progesterone combinations. On follow-up visits, her serum PTH level gradually decreased to 72.5–94.7 pg/ml, her serum TSH level reached 6.6~ 8.4 μIU/mL, and her 24-h urinary calcium was 1.8 mmol/day (RR, 2.5–7.5 mmol/day).

**Table 1:** Biochemical characteristics of the patient and her mother

	Patient	Patient’s mother	RR
Calcium (mmol/L)	2.33	2.67	2.2–2.7
Phosphate (mmol/L)	1.44	1.25	0.85–1.51
PTH (pg/mL)	201.5	42.3	15–65
25(OH)D3 (ng/mL)	26.27	23.1	20–100
GH (ng/mL)	0.13	0.18	0.12–7.7
TSH (μIU/mL)	12.64	2.6	0.5–4.3
Free T_4_ (pmol/L)	15.43	13.1	12.6–21.0
Free T_3_ (pmol/L)	4.1	5.3	3.9–7.7
LH (mIU/mL)	16.24	4.86	2.4–12.6
FSH (mIU/mL)	15.4	10.5	3.5–12.5
Estradiol (pmol/L)	60.59	232.4	45.5–854
Cortisol (8 am) (μg/dL)	16.04	14.21	6.2–19.4
ACTH (8 am) (pg/mL)	83.72	19.4	7.2–63.3
Fasting glucose (mmol/L)	4.9	4.8	3.9–6.1
Fasting insulin (μIU/mL)	20.1	16.9	2.6–24.9

No consanguinity was found for her parents. Her father showed normal appearance and average adult height (165 cm). Her mother was diagnosed with pseudopseudohypoparathyroidism (PPHP) and showed features of AHO, such as round face, short stature (140 cm), and slight brachymetatarsia (Fig. 2e,f), but no biochemical abnormalities (Table 1). Blood samples were collected from the patient, her family members, and 100 healthy Chinese controls (56 women and 44 men, age 36.1 ± 9.8 years).

This study was approved by the Institutional Ethics Committee of The Third Xiangya Hospital. Written informed consent was obtained from all subjects enrolled in this study, who agreed to join this study, with the intent of using the medical data for scientific research and publication.

### Mutation analysis

Genomic DNA was extracted from peripheral blood leukocytes by standard phenol–chloroform procedures. All 13 exons and adjacent exon–intron sequences of the *GNAS* gene (GenBank accession number NM_000516.4) were amplified by polymerase chain reaction (PCR) using the primers listed in Additional file 1: Table S1. Mutations were identified by direct sequencing of PCR products on an ABI 3730xl automated sequencer (Applied Biosystems, USA).

### Cell transfection and stimulation

To determine the phenotypic effects of the genetic mutation, we performed site-directed mutagenesis in an in vitro assay. The wild-type human GNAS pcDNA3.1 plasmid (Genechem, China) containing Asn in codon 239 of the Gsα protein was designated as Gsα-wild-type (WT), while the mutant containing Asp in the same position was designated as Gsα-239D and prepared using a Site-Directed Mutagenesis Kit (Stratagene). The WT human *GNAS* plasmid (Genechem, China) was used as the template for PCR. The primers used for PCR included: 5’-TCCAGTGCTTCGACGATGTGACTGCCATCATC-3′ and 5’-GTCACATCGTCGAAGCACTGGATCCACTTGC-3′. The fidelity of constructs was verified by restriction digestion and DNA sequencing.

For transfection experiments, we used the opossum kidney (OK) cell line (Shanghai Institutes for Biological Sciences, China) [12], and these cells have several important characteristics of proximal tubules [13, 14], including a PTH-stimulatable adenylate cyclase capacity [14, 15]. The cells were seeded in 24-well plates, maintained in minimal essential medium (MEM) and 10% fetal bovine serum at 37 °C in a 5% CO_2_ humidified atmosphere. After 24 h, the cells were transfected with 1 μg/well of WT Gsα (pcDNA3.1-GNAS WT), Gsα-239D (corresponding to the p.N239D mutant of Gsα), or control (pcDNA3.1) construct using Lipofectamine™ 2000 (Invitrogen, USA). The transfected OK cells were stimulated with 10^− 8^ M PTH 1–34 (ProSpecBio, Rehovot, Israel) or forskolin (10 μM, Abcam, UK) for 1 h. The concentration of cAMP after PTH- or forskolin-stimulation was measured using an enzyme-linked immunosorbent assay (ELISA) kit (Elabscience Biotechnology, China) following the manufacturer’s protocol. Three independent experiments were performed.

### Western blot analysis

For Western blot analysis, OK cells were transiently transfected with Gsα-WT and mutant Gsα-239D constructs. Forty-eight hours after transfection, 50 μg of cellular protein extracts from the OK cells were separated on a 10% sodium dodecyl sulfate (SDS)-polyacrylamide gel and then electroblotted onto polyvinylidene difluoride (PVDF) membranes (Millipore, USA). After blocking with 5% defatted, dried milk solution, the membranes were incubated with GNAS antibody (1:100 dilution, Abcam) for 1 h at room temperature. Subsequently, the membranes were incubated with horseradish peroxidase (HRP)-conjugated goat anti-rabbit IgG (1:5000 dilution, KPL, USA) for 1 h at room temperature. Visualization of immunoreactive bands was performed with NBT-BCIP substrate (Promega, USA).

## Results

### Detection of a novel mutation in the patient and her mother

We performed PCR to amplify a genomic DNA fragment spanning the entire coding region and the exon–intron boundaries of the *GNAS* gene. Bidirectional sequencing of the PCR products from the patient and her mother revealed a heterozygous nucleotide change from A to G at position 715 relative to the coding DNA sequence of the *GNAS* gene (NCBI Reference Sequence: NM_000516.4, Additional file 2: Figure S1A). This change (c.715A > G) resulted in a change of the amino acid residue at 239 from Asn to Asp and was designated as p.N239D. This mutation was not found in the patient’s father or other family members (Additional file 2: Figure S1B) or in 100 healthy controls, implying that the detected sequence alteration is a disease-causing mutation, not a non-functional polymorphism. We searched the Human Gene Mutation Database (HGMD, http://www.hgmd.org/) and did not find the same mutation in any previous report, indicating that this is a novel mutation.

### Reduced activity of mutant protein

**Fig. 3:**
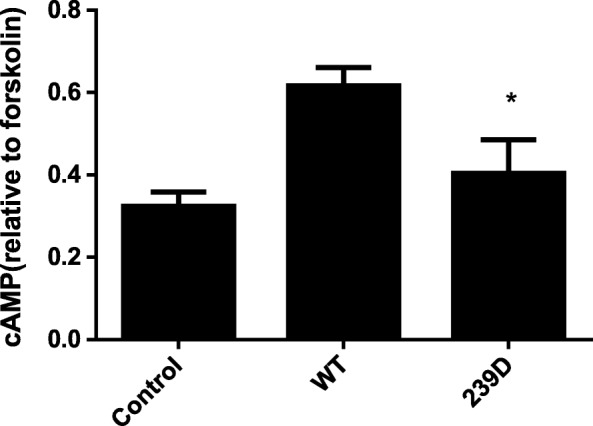
In vitro cAMP production by OK cells transfected with Gsα-wild-type (WT) or Gsα-239D (N239D, mutant). Cells transfected with the empty plasmid vector were used as controls. Compared with that in cells transfected with the WT, the PTH-induced cAMP production was significantly decreased in the Gsα-239D group when normalized to forskolin-stimulated cAMP levels. *P < 0.01

To further assess whether this novel mutation resulted in a gain or loss of function of Gsα, OK cells were transiently co-transfected with control and either the Gsα-WT or Gsα-239D mutant constructs. Quantitative analysis revealed that cells transfected with the mutant Gsα construct produced significantly less cAMP after PTH stimulation compared to those transfected with Gsα-WT when normalized to forskolin-stimulated cAMP levels (*P* < 0.01, Fig. 3), suggesting that the mutant protein had reduced Gsα activity.

### Equivalent expression of mutant and WT protein

**Fig. 4:**
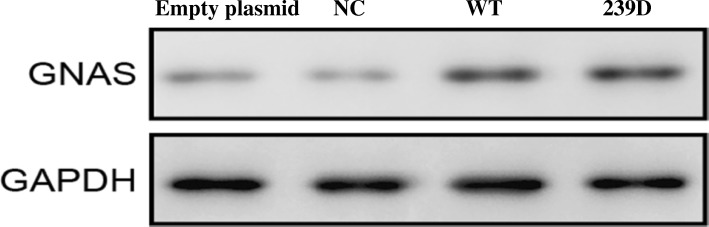
Comparison of Gsα-protein levels in OK cells transfected with WT or mutant GNAS constructs by western blot analysis. Cells transfected with the empty plasmid vector (pcDNA3.1) and non-transfected control (NC) were used as controls. The bands were blotted with anti-GNAS antibody. GAPDH was used as an internal control. The protein expression of the mutant Gsα-239D was similar to that the WT GNAS

To rule out the possibility that a difference in the expression levels of Gsα-239D and WT Gsα led to the observed difference in protein activity, we evaluated the expression levels of Gsα-239D and WT Gsα through immunoblotting analysis using a GNAS-specific antibody, and we found equivalent expression of the mutant Gsα-239D and WT Gsα (Fig. 4).

## Discussion

The manifestations of PHP1A are sometimes heterogeneous as differences in biochemical profiles and clinical features are seen among patients. Although hypocalcemia is one of the hallmarks of PHP1A, our patient had normocalcemia, a high-to-normal phosphate level, and an elevated PTH level, which is similar to several other cases [15–19]. The underlying mechanism for the normocalcemia is still unknown, but there are several hypotheses: (1) normal skeletal responsiveness to PTH might be responsible for the normocalcemia observed in some patients. For example, Ish-Shalom et al. [20] performed in vitro studies and found that the PTH receptor-coupled adenylyl cyclase system was normal in bone cells from patients with PHP1A, despite clinical evidence of impaired hormone-responsive adenylyl cyclase in other tissues, including the kidney; (2) PTH-resistance usually develops in the first few years of life in PHP1A patients, with hyperphosphatemia and elevated PTH generally preceding hypocalcemia. Thiele et al. [9] reported that 91.3% of PHP1A patients had elevated PTH levels, followed by hyperphosphatemia (50.6%) and hypocalcemia (30.9%). Thus, hypocalcemia may not be the initial presentation of a patient but is discovered during follow-up; (3) sufficient dietary intake of calcium and vitamin D may also play a role in keeping the calcium level with the normal range, as patients may have a normal or increased serum concentration of Vitamin D [16, 17]. There is still no consensus about whether PHP1A patients with normocalcemia should be treated. Some studies [1, 9] have suggested that the serum PTH in these patients should be suppressed to a normal level to protect against the skeletal demineralization effects of long-term excess PTH, which actually provided the rationale for calcitriol treatment in our patient.

In addition to PTH, many other hormones such as TSH, LH, FSH, ACTH, and growth hormone–releasing hormone (GHRH) also act via G protein-coupled (GPC) receptors, and thus, PHP1A patients may also show symptoms of insufficiency of other hormones [21–25]. As reported, subclinical hypothyroidism is among the earliest features suggestive of PHP1A and present in 70% of PHP1A cases [26]. Hypogonadism manifestations are also common in PHP1A patients who present with delayed puberty or incomplete sexual maturation. Female patients usually present with menstrual abnormalities such as primary amenorrhea/oligomenorrhea and sometimes infertility [22]. Herein, we present a case of PHP1A in a patient who had oligomenorrhea as a major symptom initially and was misdiagnosed and treated for subclinical hypothyroidism for a long time. It is known that thyroid hormones are critical for somatic growth and pubertal maturation [27, 28]. Thus, PHP1A can be confused with hypothyroidism as both diseases can manifest as short stature, obesity and menstrual disturbances. However, hypothyroidism in PHP1A is generally mild and often seen in children or adolescents who are negative for anti-thyroid antibodies and have a goiter. In clinical practice, the diagnosis of PHP1A is often delayed because some clinical features are not obvious at birth and may be very heterogeneous later. As reported [26, 29], the most common clinical features of PHP1A in toddlers are round lunar face, obesity, and subcutaneous ossifications, while other manifestations such as brachymetacarpia, seizures, subclinical hypothyroidism, and mental retardation tend to become apparent in older children. All these features are rarely present together in a given patient at the early stage of the disorder, and therefore, screening for *GNAS* mutations should be considered in patients with an atypical PHP1A phenotype.

PHP1A is caused by dominant mutations in the *GNAS* gene. Interestingly, maternal inheritance of the *GNAS* mutations leads to PHP1A, while paternal inheritance of the same mutation results in PPHP, in which features of AHO are seen but hormone resistance is not present. This imprinted pattern of inheritance can be explained by the predominantly maternal expression of Gsα in some tissues, including the renal proximal tubules [30]. In our case, we identified a novel heterozygous missense mutation c.715A > G (p.N239D) localized in exon 9 of the *GNAS* gene. The different clinical features in the daughter (PHP1A) and mother (PPHP), who shared the same mutation, indicates that the mother either has a de novo mutation involving the paternal allele or inherited this mutation from her father. To date, only four other *GNAS* mutations in exon 9 have been reported, including three missense mutations [31–34] and one insertion [35]. Therefore, the presented case involved a novel missense mutation in exon 9, which expands the spectrum of known *GNAS* mutations related to this disorder.

The Gsα protein encoded by the *GNAS* gene contains an α-helical domain and a highly conserved GTPase domain consisting of five α-helices surrounding a six-stranded β-sheet. Three flexible switch regions, I to III, connect these domains and make the guanine-binding pocket [35–38]. These regions are flexible and lead to a considerable change in structure upon GTP binding, which eventually results in the dissociation of the α-GTP complex from the βγ subunits. Switch II is supposed to govern many of the interactions of Gα with the Gβγ complex, effectors, and other secondary messengers [39]. In our patient, the novel missense mutation is located in the switch II region, which is predicted to decrease GTP binding and impair signal transduction. To evaluate whether this novel mutation resulted in a gain or loss of Gsα function, we conducted functional studies by assessing the level of cAMP activity and found a significant decrease in PTH-induced cAMP production in vitro compared to that in cells expressing WT Gsα, which strongly suggests that this mutation is associated with loss of Gsα activity. Interestingly, Farfel et al. [39] and Iiri et al. [40] described another mutation (R231H) located in the same region of exon 9 and found that the mutant Gs protein disrupts an internal salt-bridge, decreasing GTP binding and impairing signal transduction. According to their in vitro study [39], the hormone receptor-dependent stimulation of cAMP accumulation in cells expressing Gs훼-R231H is reduced by 75% in comparison to that in cells expressing Gsα-WT, which further supports that the novel *GNAS* mutation lies in a biologically important region for Gsα activity.

## Conclusion

We identified a novel *GNAS* gene mutation in a case of PHP1A with normocalcemia, which serves to further our understanding of the complex pathogenesis of PHP1A. Our results highlight the importance of a complete investigation of the *GNAS* gene in cases of suspected PHP1A when not all the classical signs are present.

## Additional files

Additional file 1: **Table S1**. PCR and sequencing primers of GNAS gene. (DOCX 14 kb)

Additional file 2: **Figure S1**. DNA sequence analysis of the exon 9 of GNAS gene. Figure S1 DNA sequence analysis of the exon 9 of GNAS gene. A The arrow indicates the novel heterozygote carrier mutation c.715A > G (p.N239D) in the proband. This mutation is also found in her mother, who was diagnosed with PPHP. B The normal sequence of her father. (DOCX 50 kb)

## Abbreviations

AHO: Albright’s Hereditary Osteodystrophy
FSH: Follicle-stimulating hormone
FT4: Free thyroxine
GNAS: Guanine nucleotide-binding protein a-stimulating polypeptide
Gsα: Stimulatory GTP binding protein
HGMD: Human Gene Mutation Database
LH: Luteinizing hormone
PCR: Polymerase chain reaction
PHP: Pseudohypoparathyroidism
PPHP: Pseudopseudohypoparathyroidism
PTH: Parathyroid hormone
TSH: Thyroid stimulating hormone
