# Supplementary material for: Identification of a novel GNAS mutation in a case of pseudohypoparathyroidism type 1A with normocalcemia
Source: BMC Med Genet. 2018 Jul 30;19:132. doi: 10.1186/s12881-018-0648-z (PMC6065144; doi:10.1186/s12881-018-0648-z)
Supplement: Supplementary file 1 — Table S1. PCR and sequencing primers of GNAS gene. (DOCX 14 kb) [file 12881_2018_648_MOESM1_ESM.docx]

Supp. Table S1 PCR and sequencing primers of *GNAS* gene

| Exon | Sense primer | PCR product(bp) | T(℃) |
| --- | --- | --- | --- |
| GNAS-1 | 5’-TCCGACCGACACCCTCCCCTTCC-3’ | 700 | 65 |
| GNAS-1 | 5’-CCCCAACACGCACCCAAACCC-3’ |  |  |
| GNAS-2 | 5’-GCAGGTAGACACTGAATTGGAC-3’ | 428 | 58 |
| GNAS-2 | 5’-GACCCTAAAGAGCCCTTCCC-3’ |  |  |
| GNAS-3 | 5’-TGGTTGAGGAATGTAGAGAGACTG-3’ | 312 | 56 |
| GNAS-3 | 5’-CTTTACTTGCTCCAAAATGTCAGG-3’ |  |  |
| GNAS-4-5 | 5’-GTGTCCTCAGGGCACATTTGG-3’ | 541 | 60 |
| GNAS-4-5 | 5’-CAGGGCTGTCACTCATGTTCC-3’ |  |  |
| GNAS-6 | 5’-GTCGGTCACATAGGGAACTCTG-3’ | 326 | 60 |
| GNAS-6 | 5’-GGGGTAACTGGTTGGCTTCTAAG-3’ |  |  |
| GNAS-7-8 | 5’-CCGTTGAGCCTGACCTTGTAG-3’ | 494 | 58 |
| GNAS-7-8 | 5’-TCCACTTGCGGCGTTCATC-3’ |  |  |
| GNAS-9-10 | 5’-CTTCGCTGCCGTGTCCTG-3’ | 581 | 58 |
| GNAS-9-10 | 5’-GCCGTGTGAATGCTTGGG-3’ |  |  |
| GNAS-11 | 5’-TCTTCAAGAGCATCTGGAACAAC-3’ | 409 | 56 |
| GNAS-11 | 5’-AGATCCTTTATGGTTTGGTGGTG-3 |  |  |
| GNAS-12 | 5’-TCCCACCACCAAACCATAAAGG-3’ | 366 | 58 |
| GNAS-12 | 5’-CTGGATGTGCGTGAACTAAAACC-3’ |  |  |
| GNAS-13 | 5’-GGTTTTAGTTCACGCACATCCAG-3’ | 467 | 58 |
| GNAS-13 | 5’-GGGGTTTCGCAAAATCACTCG-3’ |  |  |

T :annealing temperature for the PCR reactions

GNAS: guanine nucleotide-binding protein a-stimulating polypeptide
