# Supplementary material for: Identification of a novel GNAS mutation in a case of pseudohypoparathyroidism type 1A with normocalcemia
Source: BMC Med Genet. 2018 Jul 30;19:132. doi: 10.1186/s12881-018-0648-z (PMC6065144; doi:10.1186/s12881-018-0648-z)
Supplement: Supplementary file 2 — Figure S1. DNA sequence analysis of the exon 9 of GNAS gene. Figure S1 DNA sequence analysis of the exon 9 of GNAS gene. A The arrow indicates the novel heterozygote carrier mutation c.715A > G (p.N239D) in the proband. This mutation is also found in her mother, who was diagnosed with PPHP. B The normal sequence of her father. (DOCX 50 kb) [file 12881_2018_648_MOESM2_ESM.docx]

A

B

Supp. Figure S1 DNA sequence analysis of the exon 9 of *GNAS* gene. A The arrow indicates the novel heterozygote carrier mutation c.715A>G (p.N239D) in the proband. This mutation is also found in her mother, who was diagnosed with PPHP. B The normal sequence of her father.
